# Relationship of reduced glomerular filtration rate with alterations in plasma free amino acids and uric acid evaluated in healthy control and hypertensive subjects

**DOI:** 10.1038/s41598-019-46598-7

**Published:** 2019-07-16

**Authors:** M. H. Mahbub, Natsu Yamaguchi, Hidekazu Takahashi, Ryosuke Hase, Hiroshi Yamamoto, Shinya Kikuchi, Tsuyoshi Tanabe

**Affiliations:** 1 Department of Public Health and Preventive Medicine, Graduate School of Medicine, Yamaguchi University, Ube, Japan; 2 Research Institute for Bioscience Products & Fine Chemicals, Ajinomoto Co., Inc., Kawasaki, Japan

**Keywords:** Diagnostic markers, Kidney diseases

## Abstract

The potential association between altered levels of plasma free amino acids (PFAAs) and uric acid (UA) with estimated glomerular filtration rate (eGFR) remains unknown among patients with hypertension. A total of 2804 healthy controls and 2455 hypertensive patients were included in the current analysis. eGFR was defined as reduced when it was <60 ml/min/1.73 m^2^. The associations between reduced eGFR and individual PFAAs and UA in the healthy control and hypertension groups were explored by logistic regression analyses adjusted for potential confounding variables. Results show that UA had a significant positive association with reduced eGFR in both healthy control and hypertension groups (P < 0.001). Among the PFAAs, citrulline, glycine and phenylalanine showed significant positive associations with reduced eGFR in both healthy control (P < 0.01 to 0.001) and hypertension (P < 0.001) groups. Moreover, alanine, asparagine and methionine achieved significant positive associations with reduced eGFR only in the hypertension group (P < 0.01 to 0.001). Conversely, serine showed significant inverse associations with reduced eGFR in the hypertension group only (P < 0.001). Our findings provide first evidence for a strong relationship between distinct patterns of PFAAs and elevated UA with reduced eGFR in hypertension. The findings may appear useful in developing effective strategies for the prevention or early detection and treatment of declined kidney function in hypertension.

## Introduction

Kidney function decline has consistently been found to be a strong independent risk factor for adverse clinical outcomes in a broad spectrum of patients^1^. Kidney diseases with kidney function decline pose very high risks for morbidity and mortality and also enormous burdens and challenges to the global public health care system^2,3^. Published evidence suggests that kidney diseases have multidirectional relationships with hypertension and these two global health problems are projected to get worse^4,5^. For the prevention and/or better management of kidney function decline and hypertension, efforts directed towards an improved understanding of potential determinants of these two associated conditions should be of utmost importance.

Published data suggest that in chronic kidney diseases, alterations in the levels of plasma free amino acids (PFAAs) appear early and are more pronounced in advanced stages of it^6^. Also, published research works suggest the possibility of the changes in amino acid metabolism in the pathogenesis of hypertension^7,8^. Existing evidence also indicate a clear relationship of an elevated level of circulating uric acid (UA), known as hyperuricemia (HU) with the accelerated decline in kidney function^9,10^. Obermayr *et al*. suggested HU as a causal risk factor for the development and progression of chronic kidney diseases^11^. Conversely, the kidneys play major roles in the regulation of circulating UA level as they reabsorb around 90% of filtered urate and eliminate approximately 60–70% of the total UA produced in the human body^12^. On the other hand, we recently showed a clear relationship of HU with hypertension^7^; and the role of HU might be causal in the development of hypertension^13^.

From the findings of various clinical, epidemiological and experimental studies, both altered levels of PFAAs and UA appear to be independently and significantly associated with impaired kidney function which may be of particular importance among patients with hypertension. However, any such potential relationship remains unknown as no study have hitherto investigated it in the general population. Measurement of estimated glomerular filtration rate (eGFR) has long been considered the best available indicator of the overall function of the kidneys^14^. A better understanding of reduced eGFR in hypertension and its association with PFAAs and UA might contribute to better apprehension of the disease pathophysiology, and also help in the prevention or early detection and treatment of acute and chronic kidney diseases with declined kidney function in hypertension.

The purpose of the current study was to examine the differences in the concentrations of PFAAs and UA amongst apparently healthy control subjects versus hypertensive patients without and with reduced eGFR. Furthermore, we investigated the association between PFAAs and UA with reduced eGFR in both groups of subjects in an attempt to explore the existence of any relationships between alterations in PFAA and UA levels with reduced eGFR in hypertension.

## Results

### Demographic and clinical characteristics

**Figure 1:**
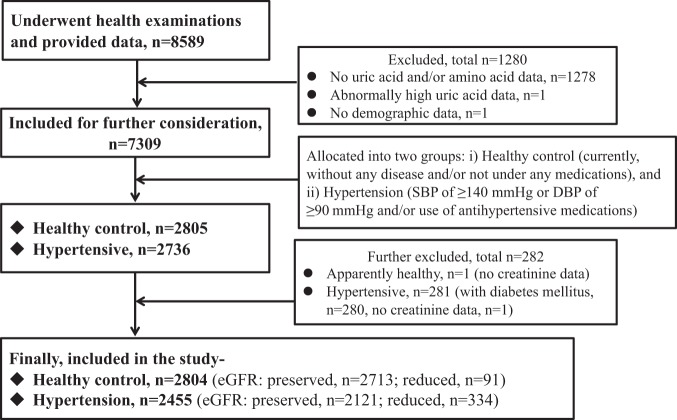
Flowchart of current study participants.

A total of 2804 subjects (1191 men, 1613 women) in the healthy control group and 2455 subjects (1300 men, 1155 women) in the hypertension group were eligible for inclusion in the analysis of this study (Fig. 1).

Table 1 shows the demographic and clinical characteristics of the study population compared between the healthy control and hypertensions groups. Compared to the subjects in the healthy control group, the patients with hypertension exhibited significantly higher values for all the demographic and clinical variables (Mann-Whitney U-test, P < 0.001) except for high-density lipoprotein cholesterol (HDLC) and eGFR, which were significantly lower in the hypertension group (Mann-Whitney U-test, P < 0.001). As shown, when compared to the healthy control group, hypertensive patients exhibited a significantly higher level of UA (median and interquartile range or IQR values were 4.6 and 1.8 mg/dL, and 5.3 and 2.0 mg/dL for healthy control and hypertension groups, respectively). Also, the latter group had a higher prevalence of HU (6.8% for controls versus 17.7 for patients). Further sex-based analysis for the prevalence of HU showed that amongst men, it was much higher than amongst women in each group: healthy controls, men 5.4% versus women 1.4% (χ^2^ test, P < 0.001); hypertension, men 12.9% versus women 4.8% (χ^2^ test, P < 0.001). Also, as shown in Table 1, hypertensive patients demonstrated a significantly declined value for eGFR compared with the healthy control group (median and IQR values were 84.9 and 21.2 mg/dl, and 74.5 and 19.4 mg/dl for healthy control and hypertension groups, respectively; Mann-Whitney U-test, P < 0.001). The prevalence of reduced eGFR in the healthy control group was 3.2% and in the hypertension group, 13.6% (χ^2^ test, P < 0.001). In the latter group, 56.7% (1371/2419; missing, n = 36) of patients were taking medications for hypertension.

**Table 1:** Demographic and clinical characteristics of the study subjects. Values are expressed as median and interquartile range (IQR).

Variables	All					Preserved eGFR					Reduced eGFR				
	Healthy control		Hypertension		P1	Healthy control		Hypertension		P2	Healthy control		Hypertension		P3
	n = 2804		n = 2455			n = 2713		n = 2121			n = 91		n = 334		
	Median	IQR	Median	IQR		Median	IQR	Median	IQR		Median	IQR	Median	IQR	
Age (Years)	44.0	26.0	65.0	18.0	<0.001	43.0	26.0	64.0	18.0	<0.001	69.0	14.0	73.0	12.3	0.003
BMI (kg/m^2^)	21.2	3.7	23.3	4.1	<0.001	21.2	3.7	23.3	4.3	<0.001	21.1	3.8	23.6	3.7	<0.001
Waist (cm)	76.0	11.5	84.0	11.0	<0.001	76.0	11.5	84.0	11.0	<0.001	78.0	13.0	84.5	10.0	<0.001
FPG (mg/dL)	91.0	10.0	97.0	13.0	<0.001	90.5	10.0	97.0	13.0	<0.001	92.0	10.5	96.0	12.0	<0.001
HbA1c (%)	5.5	0.5	5.7	0.5	<0.001	5.5	0.5	5.7	0.4	<0.001	5.7	0.3	5.8	0.4	0.005
HDLC (mg/dL)	68.0	22.0	61.0	21.0	<0.001	68.0	22.0	62.0	22.0	<0.001	66.0	22.0	57.0	20.5	<0.001
LDLC (mg/dL)	107.0	31.0	119.0	39.0	<0.001	107.0	31.0	119.0	39.0	<0.001	115.0	22.0	116.0	39.0	0.209
TG (mg/dL)	64.0	38.0	94.0	59.0	<0.001	64.0	37.0	94.0	59.5	<0.001	70.0	33.0	99.0	63.3	<0.001
SBP (mmHg)	118.0	17.0	141.0	18.0	<0.001	118.0	16.0	141.0	18.0	<0.001	122.0	15.0	140.0	20.3	<0.001
DBP (mmHg)	73.0	13.0	86.0	15.0	<0.001	73.0	13.0	87.0	13.5	<0.001	74.0	14.0	82.0	16.0	<0.001
UA (mg/dL)	4.6	1.8	5.3	2.0	<0.001	4.6	1.9	5.2	2.0	<0.001	5.5	2.0	6.0	1.9	0.029
eGFR [mL/(min × 1.73 m^2^)]	84.9	21.2	74.5	19.4	<0.001	—	—	—	—		—	—	—	—	—

eGFR, estimated glomerular filtration rate; BMI, body mass index; waist, waist circumference; FPG, fasting plasma glucose; HbA1C, haemoglobin A1C; HDLC, high-density lipoprotein cholesterol; LDLC, low-density lipoprotein cholesterol; TG, triglycerides; SBP, systolic blood pressure; DBP, diastolic blood pressure; UA, uric acid. The total number of subjects are shown under the respective columns for all variables except for waist and FPG which differs due to missing data: for all subjects, waist (control, n = 2717; hypertension, n = 2137) and FPG (control, n = 2604; hypertension, n = 2165); for subjects with preserved eGFR, FPG (control, n = 2530; hypertension, n = 1900) and waist (control, n = 2640; hypertension, n = 1885); for subjects with reduced eGFR, FPG (control, n = 74; hypertension, n = 265) and waist (control, n = 77; hypertension, n = 252). P1, P2 and P3 indicate the P-values for differences by two-tailed Mann-Whitney U-test between healthy control and corresponding hypertension groups.

We further divided both group of subjects into 2 categories each with preserved and reduced eGFR (Table 1). As revealed, the differences between the healthy control and hypertension groups remained highly significant (Mann-Whitney U-test, P < 0.01 to 0.001) for the relevant demographic and clinical variables after such categorization except for LDLC and UA for the subjects with reduced eGFR (Table 1).

### Differences in the concentrations of PFAAs

The concentrations of of PFAAs for the healthy control and hypertension groups are depicted as median and IQR values in Table 2. The concentrations of 17 out of 19 PFAAs [i.e. except asparagine (Asn) and threonine (Thr)] showed highly significant differences between those two groups of subjects (Mann-Whitney U-test, P < 0.001). Among those 17 PFAAs, the concentrations of glycine (Gly) and serine (Ser) were significantly lower and the concentrations of the rest PFAAs were significantly higher in the hypertension group than those in the healthy control group. In general, such highly significant differences between the healthy control and hypertension groups persisted among subjects with preserved eGFR. However, among subjects with reduced eGFR, such differences between the 2 groups became smaller with disappearance of the significant levels for a number of PFAAs, namely arginine (Arg), citrulline (Cit), glutamine (Gln), histidine (His), lysine (Lys), methionine (Met), ornithine (Orn), Thr, and tryptophan (Trp).

**Table 2:** Plasma free amino acid concentrations (μmol/L) in the study populations.

Amino acids	All					Preserved eGFR					Reduced eGFR				
	Healthy control		Hypertension		P1	Healthy control		Hypertension		P2	Healthy control		Hypertension		P3
	n = 2804		n = 2455			n = 2713		n = 2121			n = 91		n = 334		
	Median	IQR	Median	IQR		Median	IQR	Median	IQR		Median	IQR	Median	IQR	
Ala	310.3	94.6	345.4	105.5	<0.001	310.3	94.8	343.3	106.7	<0.001	306.6	86.5	353.9	102.9	<0.001
Arg	91.8	24.3	95.9	22.1	<0.001	91.7	24.3	95.6	21.8	<0.001	95.9	21.1	98.6	24.1	0.261
Asn	45.1	9.2	45.0	8.7	0.807	45.1	9.2	44.9	8.6	0.507	45.6	9.5	45.4	9.5	0.856
Cit	28.1	8.8	31.1	9.8	<0.001	27.8	8.5	30.2	9.1	<0.001	37.6	12.7	36.6	11.1	0.483
Gln	584.0	89.4	597.3	89.0	<0.001	583.4	90.0	596.6	88.0	<0.001	597.9	97.6	599.4	94.5	0.980
Gly	218.6	61.2	203.7	60.6	<0.001	218.2	60.7	201.5	59.3	<0.001	232.4	78.6	215.0	69.4	0.001
His	78.0	12.3	79.1	12.3	<0.001	78.0	12.3	79.3	12.3	<0.001	77.8	13.5	78.0	12.5	0.842
Ile	51.5	16.0	56.6	18.3	<0.001	51.4	16.0	56.3	18.3	<0.001	52.8	15.6	59.1	18.7	<0.001
Leu	102.6	29.3	111.5	32.6	<0.001	102.5	29.1	111.1	32.5	<0.001	103.2	29.1	113.2	32.2	0.002
Lys	175.6	43.6	186.0	40.5	<0.001	175.5	43.8	185.9	39.3	<0.001	179.4	33.0	187.7	45.3	0.038
Met	24.1	6.2	25.0	6.2	<0.001	24.1	6.2	25.0	6.2	<0.001	24.3	6.3	25.0	6.2	0.221
Orn	43.9	13.1	49.6	13.0	<0.001	43.7	13.0	49.3	12.7	<0.001	49.4	15.0	51.4	16.3	0.011
Phe	53.7	10.2	58.6	10.7	<0.001	53.5	10.1	58.0	10.6	<0.001	59.0	11.5	61.9	11.3	0.007
Pro	116.1	48.1	125.7	50.8	<0.001	116.4	48.1	124.4	50.5	<0.001	108.3	43.0	133.4	51.0	<0.001
Ser	114.9	27.3	108.0	26.0	<0.001	115.1	27.6	108.7	26.5	<0.001	106.2	24.3	102.9	22.8	0.008
Thr	120.6	33.7	120.8	34.7	0.030	120.7	33.5	121.3	34.6	0.007	109.7	39.8	116.8	34.2	0.245
Trp	51.2	11.9	53.8	11.9	<0.001	51.3	11.8	54.0	11.9	<0.001	49.6	13.4	52.0	10.9	0.013
Tyr	57.2	14.8	65.1	17.0	<0.001	57.1	14.8	64.9	17.1	<0.001	60.1	15.4	65.4	16.4	<0.001
Val	190.8	51.0	209.8	55.0	<0.001	190.7	51.1	209.2	55.1	<0.001	193.0	45.0	212.7	53.8	<0.001

Values are shown as median and and interquartile range (IQR). eGFR, estimated glomerular filtration rate. Ala, alanine; Arg, arginine; Asn, asparagine; Cit, citrulline; Gln, glutamine; Gly, glycine; His, histidine; Ile, isoleucine; Leu, leucine; Lys, lysine; Met, methionine; Orn, ornithine; Phe, phenylalanine; Pro, proline; Ser, serine; Thr, threonine, Trp, tryptophan, Tyr, tyrosine; Val, valine. P1, P2 and P3 indicate the P-values for differences by two-tailed Mann-Whitney U-test between healthy control and corresponding hypertension groups.

### Association of UA and PFAAs with reduced eGFR

The crude association of UA and individual PFAAs with reduced eGFR (no versus yes) using the logistic regression analysis has been presented in the supplementary table (Supplementary Table S1). We further evaluated those associations after adjusting the models for the relevant confounding variables (Table 3). As our adjusted results show, UA had a significant positive association with reduced eGFR in both healthy control and hypertension groups with higher odds ratio (OR) in the former group [OR 4.72, 95% CI 3.22–6.90, P < 0.001 and OR 2.42, 95% CI 1.93–3.03, P < 0.001 in healthy control and hypertension groups, respectively]. Among the PFAAs, three amino acids [Cit, Gly and phenylalanine (Phe)] demonstrated consistent relationships amongst the study population achieving significant positive associations with reduced eGFR in both the healthy control [OR between 1.53 and 2.75, 95% CI between 1.15 and 2.01 (lower) and 1.98 and 3.75 (upper), P < 0.01 to 0.001] and hypertension [OR between 1.53 and 2.35, 95% CI between 1.29 and 1.92 (lower) and 1.82 and 2.87 (upper), P < 0.001] groups. Moreover, alanine (Ala) [OR 1.46; 95% CI 1.17–1.82, P < 0.01] and Asn [OR 1.44; 95% CI 1.19–1.74, P < 0.001] demonstrated an individual positive association with reduced eGFR only in the hypertension group. On the other hand, Met demonstrated a significant positive association with reduced eGFR only in the latter group [OR 1.30, 95% CI 1.08–1.58, P < 0.01]. Conversely, Ser showed significant inverse associations with reduced eGFR in the hypertension group only [OR 0.68, 95% CI 0.54–0.85, P < 0.01]. Further analyses by logistic regression with adjustments revealed that the PFAAs showing significant positive or inverse associations with reduced eGFR also demonstrated a linear relationship across quartiles; overall, the upper quartiles entailed stronger associations compared with the corresponding lowest quartiles (Supplementary Table S2).

**Table 3:** Logistic regression analysis for the association between reduced estimated glomerular filtration rate, plasma free amino acids and uric acid in two groups of subjects with adjustment for relevant potential confounding demographic and clinical factors: healthy control group adjusted for age, sex, BMI, waist, SBP, DBP, FPG, HbA1c, LDL-C, HDL-C and TG; hypertension group adjusted for age, sex, BMI, waist, SBP, DBP, FPG, HbA1c, LDL-C, HDL-C, TG and medication.

Variables	Healthy control				Hypertension			
	(preserved, n = 2713; reduced, n = 91)				(preserved, n = 2121; reduced, n = 334)			
	OR	95% CI		P-value	OR	95% CI		P-value
		Lower	Upper			Lower	Upper	
Ala	1.01	0.69	1.49	0.958	1.46	1.17	1.82	0.001
Arg	1.16	0.81	1.67	0.426	1.24	1.01	1.52	0.042
Asn	1.15	0.83	1.57	0.401	1.43	1.19	1.74	<0.001
Cit	2.75	2.01	3.75	<0.001	2.35	1.92	2.87	<0.001
Gln	1.33	0.93	1.90	0.124	1.27	1.02	1.58	0.034
Gly	1.53	1.19	1.98	0.001	1.53	1.29	1.82	<0.001
His	1.01	0.90	1.13	0.866	0.98	0.79	1.22	0.855
Ile	1.21	0.89	1.66	0.231	1.27	1.02	1.59	0.034
Leu	1.19	0.85	1.66	0.316	1.12	0.89	1.41	0.324
Lys	1.02	0.72	1.44	0.919	1.22	0.99	1.49	0.063
Met	1.34	0.99	1.81	0.056	1.30	1.08	1.58	0.006
Orn	1.10	0.82	1.47	0.516	1.19	1.01	1.40	0.039
Phe	1.54	1.15	2.05	0.003	1.67	1.38	2.02	<0.001
Pro	0.97	0.70	1.33	0.834	1.18	0.99	1.41	0.061
Ser	0.74	0.49	1.11	0.143	0.68	0.54	0.85	0.001
Thr	0.93	0.65	1.35	0.707	0.96	0.78	1.17	0.676
Trp	0.72	0.51	1.03	0.071	0.82	0.67	1.00	0.054
Tyr	0.89	0.62	1.28	0.525	0.85	0.68	1.07	0.163
Val	0.72	0.51	1.03	0.071	0.82	0.67	1.00	0.054
UA	4.72	3.22	6.90	<0.001	2.42	1.93	3.03	<0.001

CI, confidence interval; OR, odds ratio. Ala, alanine; Arg, arginine; Asn, asparagine; Cit, citrulline; Gln, glutamine; Gly, glycine; His, histidine; Ile, isoleucine; Leu, leucine; Lys, lysine; Met, methionine; Orn, ornithine; Phe, phenylalanine; Pro, proline; Ser, serine; Thr, threonine, Trp, tryptophan, Tyr, tyrosine; Val, valine.

## Discussion

Existing literature suggests that patients with reduced eGFR are at higher risk of all-cause mortality^15^. Revealing the potential association between altered levels of PFAAs and elevated uric acid with reduced eGFR might be important in the prevention, diagnosis and management of impaired kidney function, particularly associated with hypertension. In this study, for the first time we report the association between reduced eGFR with distinct patterns of alterations in PFAAs and UA in hypertension.

In our study, significantly higher values for age and BMI for hypertensive patients simply represent the fact that the prevalence of hypertension progressively increases with age and that higher BMI is positively and significantly associated with an increased risk of hypertension^16,17^. The observations of significantly higher levels of TG and LDLC, and a lower level of HDLC in hypertension are in analogy with the existing literature as these are considered important risk factors for hypertension^7,18^. Also, patients with hypertension had a significantly higher level of FPG which is in agreement with the findings of Shen *et al*. (1988), who demonstrated that such patients, whether treated or untreated, are insulin resistant, hyperglycemic and hyperinsulinemic^18^.

In this study, the prevalence of reduced eGFR among the apparently healthy control population was 3.4%. The presence of low glomerular filtration amongst general population is not uncommon. A study conducted among adult Italians applied the same criteria for reduced eGFR as in our study and reported the age-adjusted estimates of prevalence of it as 5.7% among men and 6.2% among women^19^. Also, our finding of a significantly depressed eGFR in hypertension is in agreement with the findings of other reports showing the association of kidney function decline with an increase in blood pressure^20–22^.

HU has a clear relationship with hypertension^7^, and this has been reflected in our finding of a significantly higher level of UA among hypertensive patients. HU-induced endothelial dysfunction with impaired NO production, activation of the renin–angiotensin system with an increase in sodium resorption, promotion of vascular smooth muscle cell proliferation and arterial stiffening, increased generation of free radicals and oxidative stress, platelet adhesion and aggregation play important roles in the development of hypertension^23–25^.

Our results show that compared to the healthy control subjects, patients with hypertension had significant elevations in almost all PFAAs except for Gly and Ser (significant reductions), and Asn and Thr (non-significant changes). In a recent Japanese study, the authors found similar alterations in PFAA profiles as observed in our study when the subjects developing hypertension were compared with the subjects not developing it over a period of 4 years^8^. As observed in our study, the concentrations of Ala, isoleucine (Ile), leucine (Leu), Phe, proline (Pro), tyrosine (Tyr) and valine (Val) persisted to be significantly higher, and Gly and Ser, significantly lower in the hypertensive group than in the healthy control group, when the PFAAs were further compared among subjects with declined kidney functions. In the literature, such alterations in the concentration of PFAAs have been extensively investigated among patients with renal impairments. Chuang *et al*. (2006) compared PFAAs including all amino acids investigated in our study between pre-hemodialysis patients and age- and sex-matched healthy controls, and observed a significant increase for majority of the PFAAs including Gly in the former group (P < 0.05)^26^. In another study, compared to normal values, patients with moderate renal failure (average creatinine clearance, 32–88 ml/min) showed a significant increase in Ala, Cit, Gly, Lys, Orn, Phe, Pro and Val (P < 0.005 to 0.001); conversely, Arg and Ser showed a decreasing trend although not significant^27^. Ceballos *et al*. (1990) compared the patterns of PFAA concentrations between healthy controls and patients with mild renal failure (creatinine clearance >25 ml/min) and exhibited significantly raised concentrations of Cit and Orn and low level of Ser (P < 0.001) amongst the patients^28^. Overall, our findings for alterations in PFAAs among subjects with reduced eGFR corroborate with those of other relevant studies. However, we compared PFAAs between the hypertensive group without any diagnosed kidney diseases and the control group, both with reduced eGFR; conversely, other studies included patient groups with advanced kidney diseases and the control group with preserved kidney functions. All these might have led to the observed differences in PFAAs between the studies.

In the present study, we confirmed the association between PFAAs and UA with reduced kidney function in healthy control subjects and hypertensive patients after adjustments for potential relevant confounders. However, compared to the healthy control group, the modest attenuation in OR for the positive association of UA with reduced kidney function in hypertension was probably caused by the fact that UA level represented a similar dimension (higher levels of UA) for both preserved and declined categories of patients in hypertension. Furthermore, as revealed in this study, Cit, Gly and Phe were positively associated with reduced eGFR in both healthy control and hypertensive groups (Supplementary Table S3). In human body, the kidneys play major roles in the production of these PFAAs: they take up Cit, Gly and Phe from bloodstream and convert them to Arg, Ser and Tyr, respectively and release into renal veins^29^. Therefore, it might be possible that reduced kidney function causes diminished renal uptake of Cit, Gly and Phe and decreased production of Arg, Ser and Tyr, and thus leads to a higher level of former amino acids in plasma. On the other hand, significant positive associations of Ala, Asn and Met, and a significant inverse association of Ser with reduced eGFR were revealed in hypertension only (Supplementary Table S3). The role of kidney function decline on plasma levels of these PFAAs cannot be explained from the scarce relevant literature. However, the association of Ala, Asn and Met with hypertension has been clearly stated in the literature. In the INTERMAP epidemiological study, a strong association of urinary excretion of Ala with higher blood pressure amongst the participants was demonstrated by applying metabolic phenotyping of the collected data^30^. Another amino acid Met, which is a precursor of homocysteine can inhibit the production of endothelial nitric oxide (NO) via accumulation of asymmetric dimethylarginine (ADMA), a competitive inhibitor of the latter, and thereby cause an elevation in blood pressure^31^. Also, a prior study demonstrated that intakes of Ala and Met were positively associated with higher blood pressure among patients with prevalent cardiovascular diseases^32^. In animal experiments, intracisternal injections of L-Asn produced pressor responses in the freely moving rat^33^. In contrast, Ser was negatively associated with declined eGFR in hypertension. The amino acid Ser is produced from Gly in the kidneys^29^. As our results show, the existing concentration of Gly was significantly lower in hypertension causing less availability of it for the production of Ser. Furthermore, persistent reduced ability of the kidneys to produce Ser probably made its plasma concentration low.

**Figure 2:**
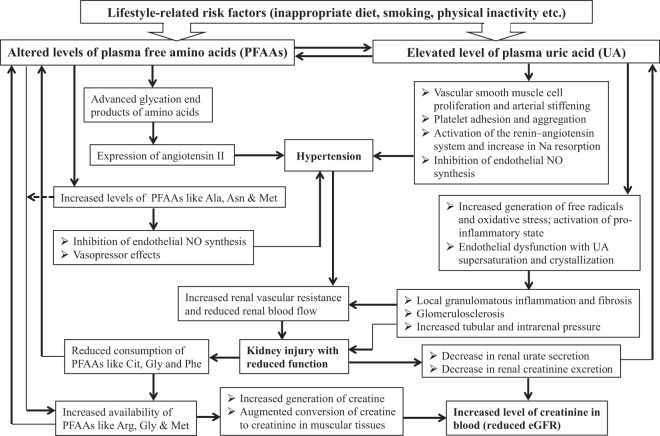
Schematic diagram showing the probable roles of altered concentrations of plasma free amino acids and uric acid in reduced eGFR with respect to hypertension.

Our study design does not allow us to point out whether the observed changes in the levels of PFAA and UA in hypertension were a cause or merely a consequence of reduced kidney functions, and vice versa. However, it appears that close, complex and multidirectional links possibly exist between the altered levels of PFAAs and UA with hypertension and reduced eGFR. Based on our results mentioned above and relevant discussion presented here, a hypothetical model of those relationships has been presented schematically in Fig. 2. Certain amino acids take part in the biosynthesis of purine and subsequent formation of UA^34^. Alterations in PFAA levels triggered by lifestyle-related factors possibly induce an increase in plasma UA which in turn promotes further alterations in the levels of PFAAs. Such an interrelationship between amino acids and UA has been mentioned as the amino-uric interaction^7^. Thus, altered levels of PFAAs and HU probably induce an elevation in blood pressure which increases renal vascular resistance with subsequent reductions in renal blood flow followed by a decrease in renal urate secretion in the proximal tubule, causing a further increase in the preexisting elevated level of plasma UA^25,35^. On the other hand, kidneys play crucial role in the production of a number of important amino acids^29^. Also, certain PFAAs and renal functional status influences excretion of creatinine by kidneys^36,37^. Conversely, parenteral and oral supplementation of selective amino acids has been reported to be associated with the arrest of the progression of renal failure, renal repair and amelioration of its functional insufficiency^38,39^. All these observations suggest the existence of a possible relationship of PFAAs and UA with functional status of the kidneys (Fig. 2).

There are several potential limitations to this study. First, this study was conducted amongst the Japanese population, and hence, generalization of study findings may be limited. Second, we did not assess background information such as diet, smoking status or alcohol consumption etc. However, we think that any such effects on our study findings might be limited as we included a control group and the logistic regression models were generated separately for both the control and hypertension groups. Third, we did not collect data on uric acid-lowering medications. However, we firmly believe that this did not influence the outcome of our study as the control group was not under any medications and the use of such medications in the hypertension group would mean an underestimation of current results. Fourth, the cross-sectional design of our study does not allow any speculation on causality or temporality for any associations observed here. Fifth, we did not include the measurement of taurine, a PFAA that influences different physiologic and biologic processes in the kidney and may serve as a potential biomarker of kidney injury in various pathological conditions. Nonetheless, we believe that this does not affect the interpretation of our current results as the inclusion of the measurement of taurine in our study would have probably provided additional support to the observed associations between PFAAs and reduced kidney function. However, future studies should elucidate the clinical significance of the association between any alteration in the plasma level of taurine and reduced kidney function. Another potential weakness of our study may be the fact that we did not show the differences in the levels of PFAAs between different groups of patients with hypertension categorized according to different levels of blood pressure. However, this does not influence our study findings as we presented the results of this study after adjustments for SBP and DBP along with other demographic and clinical variables. Lastly, Fig. 2 of this study schematically depicts the hypothetical associations of alterations between PFAAs and UA with reduced eGFR in hypertension that are based on the discussion of our study findings presented above and also the information available in the published literature. Therefore caution is needed when interpreting this figure as the information presented here is not based on any relevant statistical approach like mediation analysis.

For the first time, our study provides evidence for the potential existence of a close relationship between altered levels of PFAAs and UA with reduced eGFR in hypertension. In future, longitudinal studies should investigate large samples from general populations including disease-free individuals and measure the changes in PFAAs and UA over time, and confirm which changes occur earlier and also what are the effects of hypertension on decline of glomerular filtration rate or vice versa.

## Methods

### Study design and ethical issues

This cross-sectional study was conducted in Shimane Prefecture, Japan. Relevant institutional review board of Shimane University (20100129-3) and Yamaguchi University (H25-26-2) approved the study protocol which was conducted in accordance with the Declaration of Helsinki. The study participants were briefed verbally about the detailed protocol and provided written informed consent to participate in this study.

### Study population

The subject selection procedure has been described elsewhere^7^. Figure 1 depicts the flowchart of the study participants. Briefly, the study population comprised 8589 subjects who underwent annual health check-up that included physical examinations, clinical and other laboratory tests between 2010 and 2012. Also, data on personal and medical history were collected using a self-administered questionnaire. According to predefined criteria, subjects were selected for the healthy control (n = 2805) and hypertension (n = 2736) groups. The control group comprised apparently healthy subjects, and currently, they were not taking any medications. Further exclusion (including 282, mainly due to the coexistence of diabetes mellitus or DM in hypertension) of subjects left 2804 subjects in the control group and 2455 subjects in the hypertension group for final inclusion in this study. The hypertensive patients were free from any serious health problems such as cancer or renal failure.

### Measurement of clinical and laboratory variables

During the check-up, venous blood samples were collected from the cubital vein of seated subjects after an 8-hour overnight fast. Fasting plasma glucose (FPG) and hemoglobin A1c (HbA1c) were determined using the hexokinase method and latex agglutination immunoassay, respectively. The concentrations of high-density lipoprotein cholesterol (HDLC), low-density lipoprotein cholesterol (LDLC), and triglyceride (TG) in the serum were measured enzymatically. Plasma UA level was measured using the uricase-HMMPS method by L-type UA.M kit (Wako Pure Chemical Industries, Ltd., Osaka, Japan). Serum concentration of creatinine was measured by using the creatininase-HMMPS method following the manufacturer’s instructions (Wako Pure Chemical Industries, Osaka, Japan).

In this study, we determined the absolute concentrations (in μmol/L) of 19 amino acids: Ala, Arg, Asn, Cit, Gln, Gly, His, Ile, Leu, Lys, Met, Orn, Phe, Pro, Ser, Thr, Trp, Tyr, and Val. We did not attempt to measure other genetically encoded amino acids like glutamate, aspartate, and cysteine due to their known instability in the blood^7,8^. Measurements of PFAA concentrations were performed by high-performance liquid chromatography–electrospray ionization–mass spectrometry (HPLC–ESI–MS) followed by precolumn derivatization which allows such measurements with high accuracy. For this, we followed the protocol as described elsewhere^40,41^. Briefly, 5 ml of blood samples were collected into tubes containing ethylenediaminetetraacetic acid (EDTA; Terumo Co. Tokyo, Japan), which were put on ice immediately and kept for about 15 min. Then the tubes were centrifuged at 4 °C (3,000 rpm, 15 min) and the plasma was separated into tubes. These tubes were stored at −80 °C for a period 2 weeks to 2 months until the analysis for PFAAs was performed.

### Clinical assessments

We defined hypertension as a systolic blood pressure (SBP) ≥ 140 mmHg or a diastolic blood pressure (DBP) ≥ 90 mmHg and/or the use of antihypertensive medication/s; DM as a FPG ≥ 126 mg/dL, a HbA1c ≥ 6.5%, and/or the use of medication/s for DM [Mahbub *et al*. 2017]. We defined HU as a plasma UA ≥ 7 mg/dL in males and ≥6.0 mg/dL in females^42,43^.

In this study, eGFR was calculated using the serum creatinine data for each subject, applying the following equation recommended for the Japanese population by the Japanese Society of Nephrology: eGFR (mL/min/1.73 m^2^) = 194 × Serum creatinine (−1.094) × Age (−0.287) × 0.739 (if female)^44^. Subsequently, eGFR was classified into two categories: (1) preserved eGFR (eGFR ≥ 60 ml/min/1.73 m^2^) and (2) reduced eGFR (eGFR < 60 ml/min/1.73 m^2^)^45,46^.

### Statistical analyses

The continuous variables of this study were presented as the median and interquartile range (IQR). The differences for demographic and clinical variables between the two groups were assessed by the Mann-Whitney U-test for the continuous variables, and by the Chi-square (χ^2^) test for the categorical variables. We explored the association between reduced kidney function and individual amino acids and UA in each group by the logistic regression analyses. At first, we investigated the crude association between the independent variables and the outcome variable by the logistic regression analyses. For this, the values of all PFAAs and UA were scaled to multiples of 1 IQR calculated separately for males and females as significant sex-differences exist for these values^9,47^. Next, we adjusted the logistic regression models for the potential confounding factors showing significant group differences with additional inclusion of use of antihypertensive medications (yes, no) in the hypertension group. Furthermore, to clarify the direction of the relationships between the PFAAs showing significant associations with reduced kidney function, the former variable were divided into quartiles and logistic regression analyses were conducted with adjustments for the same potential confounders. From the logistic regression analyses, we obtained the odds ratios (OR) for UA and individual amino acids with corresponding 95% confidence interval (CI) and P-values. A two-tailed P < 0.01 was considered significant. The statistical analyses were performed with the software package SPSS version 22 for Windows (SPSS Inc., Chicago, IL, USA).

## Supplementary information

Supplementary Information

## Data Availability

Requests for data and materials should be addressed to the corresponding author.
