# Supplementary material for: Relationship of reduced glomerular filtration rate with alterations in plasma free amino acids and uric acid evaluated in healthy control and hypertensive subjects
Source: Sci Rep. 2019 Jul 16;9:10252. doi: 10.1038/s41598-019-46598-7 (PMC6635408; doi:10.1038/s41598-019-46598-7)
Supplement: Supplementary file 1 — Supplementary Information [file 41598_2019_46598_MOESM1_ESM.pdf]

Relationship of reduced glomerular filtration rate with alterations in plasma free amino acids and uric acid evaluated in healthy control and hypertensive subjects

MH Mahbub[1], Natsu Yamaguchi[1], Hidekazu Takahashi[1], Ryosuke Hase[1], Hiroshi Yamamoto[2], Shinya Kikuchi[2], Tsuyoshi Tanabe[1]

[1]Department of Public Health and Preventive Medicine, Graduate School of Medicine, Yamaguchi University, Japan.

[2]Research Institute for Bioscience Products & Fine Chemicals, Ajinomoto Co., Inc., Japan.

	Healthy control				Hypertension			
	(preserved, n = 2713; reduced, n = 91)				(preserved, n = 2121; reduced, n = 334)			
		95% CI				95% CI		
Variables	OR	Lower	Upper	P-value	OR	Lower	Upper	P-value
Ala	0.96	0.73	1.26	0.771	1.24	1.08	1.43	0.002
Arg	1.26	0.99	1.60	0.056	1.15	0.99	1.33	0.061
Asn	1.10	0.89	1.36	0.368	1.18	1.04	1.34	0.012
Cit	2.74	2.28	3.29	<0.001	2.84	2.45	3.30	<0.001
Gln	1.44	1.15	1.81	0.002	1.11	0.98	1.25	0.115
Gly	1.30	1.09	1.56	0.005	1.27	1.15	1.41	<0.001
His	1.00	0.88	1.14	0.970	0.86	0.74	0.99	0.038
Ile	1.11	0.88	1.41	0.376	1.23	1.06	1.43	0.006
Leu	1.13	0.88	1.47	0.343	1.03	0.88	1.20	0.743
Lys	1.16	0.92	1.48	0.215	1.08	0.93	1.25	0.335
Met	1.23	0.96	1.58	0.105	1.05	0.92	1.21	0.464
Orn	1.38	1.17	1.63	<0.001	1.36	1.23	1.51	<0.001
Phe	1.91	1.59	2.29	<0.001	1.59	1.41	1.81	<0.001
Pro	0.87	0.67	1.12	0.281	1.16	1.03	1.32	0.017
Ser	0.63	0.47	0.85	0.002	0.67	0.58	0.79	<0.001
Thr	0.83	0.68	1.02	0.080	0.77	0.67	0.90	0.001
Trp	0.73	0.56	0.96	0.023	0.72	0.62	0.83	<0.001
Tyr	1.37	1.10	1.71	0.006	0.98	0.85	1.14	0.823
Val	1.00	0.99	1.01	0.977	1.04	0.89	1.20	0.637

UA	2.94	2.29	3.76	<0.001	1.90	1.65	2.19	<0.001

Table S1. Logistic regression for the crude association (without adjustments) between reduced estimated glomerular filtration rate, plasma free amino acids and uric acid in two groups of subjects. CI, confidence interval; OR, odds ratio. Ala, alanine; Arg, arginine; Asn, asparagine; Cit, citrulline; Gln, glutamine; Gly, glycine; His, histidine; Ile, isoleucine; Leu, leucine; Lys, lysine; Met, methionine; Orn, ornithine; Phe, phenylalanine; Pro, proline; Ser, serine; Thr, threonine; Trp, tryptophan; Tyr, tyrosine; Val, valine.

		Healthy control				Hypertension			
		(n: preserved, n = 2713; reduced, n = 91)				(n: preserved, n = 2121; reduced, n = 334)			
			95% CI		P-value		95% CI		P-value
Variables	Quartiles	OR	Lower	Upper		OR	Lower	Upper	
Ala	1	Reference	–	–	–	Reference	–	–	–
	2	0.77	0.36	1.64	0.500	1.50	0.91	2.45	0.110
	3	0.78	0.36	1.66	0.519	1.89	1.15	3.10	0.011
	4	0.91	0.42	1.98	0.803	1.96	1.15	3.33	0.014
Asn	1	Reference	–	–	–	Reference	–	–	–
	2	1.05	0.47	2.35	0.899	1.30	0.77	2.19	0.331
	3	1.49	0.66	3.37	0.333	1.90	1.15	3.13	0.012
	4	1.61	0.70	3.72	0.263	2.31	1.40	3.81	0.001
Cit	1	Reference	–	–	–	Reference	–	–	–
	2	4.72	0.56	39.86	0.154	2.55	1.26	5.18	0.010
	3	12.75	1.66	98.20	0.015	4.81	2.45	9.45	<0.001
	4	21.41	2.77	165.66	0.003	8.03	4.12	15.68	<0.001
Gly	1	Reference	–	–	–	Reference	–	–	–
	2	1.51	0.62	3.68	0.360	2.07	1.17	3.66	0.013
	3	2.53	1.02	6.27	0.044	3.88	2.10	7.16	<0.001
	4	3.77	1.54	9.20	0.004	4.70	2.55	8.65	<0.001
Met	1	Reference	–	–	–	Reference	–	–	–
	2	1.03	0.45	2.37	0.950	1.54	0.95	2.49	0.081
	3	1.33	0.61	2.92	0.478	1.63	0.99	2.68	0.053

	4	1.65	0.77	3.53	0.199	1.92	1.19	3.10	0.008
Phe	1	Reference	–	–	–	Reference	–	–	–
	2	0.55	0.16	1.88	0.345	2.19	1.23	3.91	0.008
	3	1.69	0.63	4.57	0.298	2.75	1.56	4.87	<0.001
	4	2.30	0.87	6.11	0.093	4.26	2.42	7.49	<0.001
Ser	1	Reference	–	–	–	Reference	–	–	–
	2	1.47	0.75	2.85	0.259	0.79	0.51	1.22	0.288
	3	0.43	0.18	1.04	0.062	0.75	0.48	1.18	0.213
	4	0.79	0.35	1.81	0.579	0.45	0.27	0.74	0.002

Table S2. Logistic regression analysis for the association between reduced estimated glomerular filtration rate, and quartiles of selected plasma free amino acids in two groups of subjects with adjustment for relevant potential confounding demographic and clinical factors: healthy control group adjusted for age, sex, BMI, waist, SBP, DBP, FPG, HbA1c, LDL-C, HDL-C and TG; hypertension group adjusted for age, sex, BMI, waist, SBP, DBP, FPG, HbA1c, LDL-C, HDL-C, TG and medication. CI, confidence interval; OR, odds ratio. Ala, alanine; Asn, asparagine; Cit, citrulline; Gly, glycine; Met, methionine; Phe, phenylalanine; Ser, serine.

**Amino acid**	**Association with reduced kidney function**			
	Healthy control		Hypertension	
	Yes/No	Type	Yes/No	Type
Cit	Yes	Positive	Yes	Positive
Gly	Yes	Positive	Yes	Positive
Phe	Yes	Positive	Yes	Positive
Ala	No		Yes	Positive
Asn	No		Yes	Positive
Met	No		Yes	Positive
Ser	No		Yes	Negative

Table S3. Summary of observed association between reduced kidney function and plasma free amino acids and the type of association revealed in logistic regression analysis with adjustments for potential confounders in healthy control and hypertension groups. Ala, alanine; Asn, asparagine; Cit, citrulline; Gly, glycine; Met, methionine; Phe, phenylalanine; Ser, serine.
